# Genetic interaction mapping of Aurora protein kinases in mouse oocytes

**DOI:** 10.3389/fcell.2024.1455280

**Published:** 2024-09-25

**Authors:** Cecilia S. Blengini, Karen Schindler

**Affiliations:** ^1^ Department of Genetics, Rutgers, The State University of New Jersey, New Brunswick, NJ, United States; ^2^ Human Genetics Institute of New Jersey, New Brunswick, NJ, United States

**Keywords:** oocyte, meiosis, aneuploidy, aurora kinase, mouse model

## Abstract

The Aurora Kinases (AURKs) are a family of serine-threonine protein kinases critical for cell division. Somatic cells express only AURKA and AURKB. However, mammalian germ cells and some cancer cells express all three isoforms. A major question in the field has been determining the molecular and cellular changes when cells express three instead of two aurora kinases. Using a systematic genetic approach involving different Aurora kinase oocyte-specific knockout combinations, we completed an oocyte-AURK genetic interaction map and show that one genomic copy of *Aurka* is necessary and sufficient to support female fertility and oocyte meiosis. We further confirm that AURKB and AURKC alone cannot compensate for AURKA. These results highlight the importance of AURKA in mouse oocytes, demonstrating that it is required for spindle formation and proper chromosome segregation. Surprisingly, a percentage of oocytes that lack AURKB can complete meiosis I, but the quality of those eggs is compromised, suggesting a role in AURKB to regulate spindle assembly checkpoint or control the cell cycle. Together with our previous studies, we wholly define the genetic interplay among the Aurora kinases and reinforce the importance of AURKA expression in oocyte meiosis.

## Introduction

The Aurora Kinases (AURKs) are a family of serine-threonine protein kinases critical for cell division, in both mitosis and meiosis (Nguyen and Schindler, 2017). Three *Aurk* isoforms are encoded in the mammalian genome: Aurora kinase A (AURKA), Aurora kinase B (AURKB), and Aurora kinase C (AURKC). Somatic cells express only AURKA and AURKB. In somatic cells, AURKA localizes to centrosomes and is important for centrosome duplication and separation; whereas AURKB is the catalytic subunit of the chromosomal passenger complex (CPC), which localizes to centromeres and contributes to chromosome alignment and spindle assembly checkpoint (SAC) activation (Carmena and Earnshaw, 2003). However, mammalian germ cells express all three isoforms. A major question in the field has been determining how the molecular and cellular contexts change when cells express three, instead of two, aurora kinases.

Studies by us, and others, demonstrate that these proteins specialize to perform different functions and regulate each other in a complex manner in mouse oocytes (Balboula and Schindler, 2014; Blengini et al., 2021a; Nguyen et al., 2018; Sharif et al., 2010; Solc et al., 2012; Vallot et al., 2018). For example, in oocytes AURKC replaces AURKB as the catalytic subunit of the CPC, where it localizes at the inter-chromatid axis, kinetochores, and spindle poles (Balboula et al., 2016; Schindler et al., 2012), and is required for chromosome alignment (Schindler et al., 2012) and bipolar spindle formation (Balboula et al., 2016; Blengini et al., 2024). As a result of AURKC functioning on chromosomes, AURKB appears displaced in the cytoplasm concentrating around the spindle (Balboula and Schindler, 2014). Through oocyte-specific deletion, data indicates that its role is to maintain oocyte quality during maternal aging (Nguyen et al., 2018). In these oocytes, there was reduced SAC protein expression, leading to a premature increase in egg aneuploidy with increasing maternal age (Blengini et al., 2021b). Importantly, both AURKA and AURKC activities are increased in *Aurkb* knockout oocytes, suggesting that some phenotypes could be driven by unbalanced AURKA/C functions (Aboelenain and Schindler, 2021; Nguyen et al., 2018). Although AURKA predominately localizes to spindle poles, there is evidence of a chromosome-localized AURKA sub-population (Kratka et al., 2022), similar to somatic cells (DeLuca et al., 2018). Females in which AURKA was specifically deleted in oocytes are sterile because AURKA is required for several spindle assembly steps. First, AURKA controls acentrosomal microtubule organizer center (aMTOC) fragmentation and helps organize a liquid-like spindle domain (LISD). Second, AURKA ensures proper MI spindle length, and maintains the LISD. Third, AURKA is required for spindle stability (Blengini et al., 2021a; Blengini et al., 2024). Surprisingly, females whose oocytes express only AURKA (BC-knockout, KO), are fertile. In these BC-KO oocytes, AURKA compensates for AURKB/C absence (Nguyen et al., 2018). Further analysis revealed that AURKA specifically compensates for AURKC, and that AURKC competition for CPC binding ensures that AURKA can efficiently regulate MI spindle length. Therefore, the three AURKs have complex genetic interactions.

These complex interactions were determined through comparing single and double *Aurk* KO oocytes, but, to date, we have not analyzed all potential genetic combinations. To dissect the specific contributions of each AURK, we completed generation of mouse models lacking the missing *Aurk* KO combinations and performed phenotypic analyses on these animals and their oocytes. Through these analyses, we demonstrate that all strains lacking *Aurka* are sterile, only one genomic copy of *Aurka* is necessary and sufficient for female fertility and spindle formation, and that expression of only AURKC can drive cytokinesis and polar body extrusion in the presence of severe chromosome misalignment.

## Materials and methods

### Mouse strains

The different strains of mice used in this work were generated by mating floxed *Aurka* (C57BL/6N) (*Aurka* ^f/f^) mice or *Aurka* ^fl/fl^ Gdf9-Cre mice with double knockout mice for AURKB and AURKC (C57BL/6J, 129/Sv) (*Aurkb* ^f/f^, *Aurkc* ^−/−^, Gdf9-Cre mice (BC-KO), described before (Blengini et al., 2021a; Nguyen et al., 2018; Schindler et al., 2012). After several rounds of mating, we isolated animals that were *Aurka* ^f/f^, *Aurkb* ^f/f^, and Gdf9-Cre mice (AB-KO), *Aurka* ^f/f^, *Aurkc* ^−/−^, and Gdf9-Cre mice (AC-KO), *Aurka* ^f/f^, *Aurkb* ^f/f^, *Aurkc* ^−/−^, and Gdf9-Cre mice (ABC-KO) and *Aurka* ^f/+^, *Aurkb* ^f/f^, *Aurkc* ^−/−^, Gdf9-Cre mice (A-Het, BC-KO). Moreover, we also used animals *Aurkb* ^f/f^, *Aurkc* ^−/−^, and Gdf9-Cre mice (BC-KO). Control animals (Wild-type (WT)) were *Aurka* ^f/f^, and *Aurkb* ^f/f^ but lacked the Cre recombinase transgene. Mice were housed on a 12–12 h light-dark cycle, with constant temperature and with food and water were provided *ad libitum*. Animals were maintained in accordance with guidelines of the Institutional Animal Use and Care Committee of Rutgers University (protocol 201702497) and the guidelines of National Institutes of Health guidelines. All oocyte experiments were conducted using healthy female mice aged 6–16 weeks.

### Genotyping

Genotyping was performed before weaning and repeated upon use of the animals for experiments for replication and confirmation. LoxP and Cre genotyping was performed by PCR amplification. Primers for *Aurka LoxP* (Forward: 5′-CTG​GAT​CAC​AGG​TGT​GGA​GT-3′, Reverse: 5′-GGC​TAC​ATG​CAG​GCA​AAC​A-3′), *Aurkb LoxP* (Forward: 5′-AGG​GCC​TAA​TTG​CCT​CTT​GT-3′, Reverse: 5′-GGG​CAT​GAA​TTC​TTG​AGT​CG-3′), and *Gdf9-Cre* (Forward: 5′-GGC​ATG​CTT​GAG​GTC​TGA​TTA​C-3′, Reverse: 5′-CAG​GTT​TTG​GTG​CAC​AGT​CA-3′, Internal control Forward: 5′-CTA​GGC​CAC​AGA​ATT​GAA​AGA​TCT-3′, Internal control Reverse: 5′-GTA​GGT​GGA​AAT​TCT​AGC​ATC​ATC​C-3′). Primers were used at a final concentration of 1 μM, and Taq 2xMaster Mix (NEB, #M0270L) was used following the manufacturer’s protocol. The sizes of products were: *Aurka*: 243 bp for WT and 372 bp for lox/lox transgene; *Aurkb*: 350 bp for WT and 500 bp for lox/lox transgene, for Cre: 324 bp for Cre absence and 200 bp + 324 bp for Cre presence. Deletion of *Aurkc* was detected by a TaqMan copy number assay using primers/probes to detect Neo (Assay #Mr00299300_cn) and Tfrc (for normalization; Assay #4458366). The delta-delta Ct method was used to determine expression levels. PCR conditions are available upon request.

### Fertility trials

The number of pups per litter was recorded from matings where sexually mature WT, AB-KO, AC-KO, Het A BC-KO, and ABC-KO females at 6–8 weeks of age were mated with fertile wild-type B6D2 (Jackson Laboratories B6D2F1/J, #100006) males until a total of 6 litters were produced by WT females, approximately 7 months.

### Oocyte collection and maturation

Prophase I-arrested oocytes were collected from ovaries of 6–16 weeks old females injected 48 h earlier with 5 I.U. of pregnant mare’s serum gonadotropin (PMSG) (Lee Biosolutions #493–10). Oocytes were collected as described previously (Blengini and Schindler, 2018). Briefly, ovaries were placed in minimal essential medium (MEM) containing 2.5 μM milrinone (Sigma-Aldrich #M4659) to prevent meiotic resumption and oocytes were isolated by piercing the ovaries with needles. To induce meiotic resumption, oocytes were cultured in milrinone-free Chatot, Ziomek, and Bavister (CZB) medium in an atmosphere of 5% CO_2_ in air at 37°C. Oocytes were matured for 7–7.5 h after milrinone wash, to reach metaphase I.

### Ovulation induction

Ovulated eggs were collected from ampullas of females of 6–16 weeks of age that had been hormonally stimulated with 5 I.U. of PMSG 48 and 5 I.U. of human chorionic gonadotrophin (hCG) (Sigma-Aldrich #CG5) 12 h prior of the collection. The ampullas were dissected in MEM supplemented with 3 mg/ml Hyaluronidase (Sigma; H3506).

### Ovarian histology

After conclusion of fertility trials, ovaries were fixed in Modified Davidson’s fixative solution (Electron Microscopy Sciences, #6413–50) overnight at room temperature. Samples were processed by the pathology facility at the Office of Translational Service at Rutgers University. Five µm sections of ovaries were stained with H&E and imaged using Zeiss Axiovert 200M bright field microscope with a ×63 objective. The number of each type of follicle was assessed as previously described (Myers et al., 2004).

### Immunocytochemistry

After maturation, oocytes were immunostained according to a previously described protocol (Blengini and Schindler, 2018). Briefly, oocytes were fixed in 1X PBS containing 2% paraformaldehyde (PFA) for 20 min at room temperature. Oocytes were then incubated in permeabilization solution (PBS containing 0.1% (vol/vol) Triton X-100% and 0.3% (wt/vol) BSA) for 20 min, followed by 10 min in blocking buffer (0.3% BSA containing 0.01% Tween in PBS). Immunostaining was performed by incubating cells in primary antibody overnight in a dark, humidified chamber at 4C followed by 3 consecutive 10-min incubations in blocking buffer. After washing, secondary antibodies were diluted 1:200 in blocking solution and the sample was incubated for 1 h at room temperature. After washing, the cells were mounted in 10 μL VectaShield (Vector Laboratories, #H-1000) with 4′, 6- Diamidino-2-Phenylindole, Dihydrochloride (DAPI; Life Technologies #D1306; 1:170).

### *In Situ* chromosome spreads

Egg ploidy status was assessed as previously described (Stein and Schindler, 2011). Briefly, oocytes matured for 16h until they reached metaphase II and cultured in 100 μM Monastrol (Sigma #M8515) in an atmosphere of 5% CO_2_ in air at 37°C for 2h to destabilize the spindle and separate the chromosomes. Eggs were fixed in PBS containing 2% paraformaldehyde (PFA) for 20 min at room temperature and stained with anti-centromeric (ACA) to detect centromeres. The number of sister pairs were quantified, considering 20 pairs as a euploid egg and any deviation of that number as an aneuploid egg.

### Antibodies

Primary antibodies and concentrations were used as follows: Pericentrin (PCNT) (mouse, 1:100; BD Biosciences, #611814); TACC3 (Rabbit, 1:100; Novus Biologicals # NBP2-67671), Alpha Tubulin polyclonal (Sheep, 1:100; Cytoskeleton #ATN02), pABC, (rabbit, 1:100; Cell Signaling Technology #2914), and anti-centromeric (ACA) (Human 1/30; Antibodies Incorporated; #15–234). Secondary antibodies were used at 1:200: anti-mouse-Alexa-568 (Life Technologies #A10037), anti-human-Alexa-633 (Life Technologies #A21091), anti-rabbit-Alexa-647 (Life Technologies#A31573) and Anti-sheep-Alexa-488 (Life Technologies #A11015).

### Microscopy

Oocytes images were captured using a Leica SP8 confocal microscope equipped with 40×1.30 NA oil immersion objective. Optical z-stacks of 1.0 μm step with a zoom of 4.0. In those experiments where pixel intensity was compared, the laser power was kept constant among genotypes.

### Image analysis

For analysis of TACC3 pixel intensity in fixed oocytes, Fiji software (Schindelin et al., 2012) was used. We made a maximal projection of the z-stack and then defined a region of interest around the spindle using a free-handed drawing tool. Finally, we measured TACC3 intensity in that region of interest using the measurement tool. The volume of spindle and aMTOC fragments in fixed images was performed in Imaris software (Bitplane).

### Statistical analysis

All experiments were conducted 3 times, and any exception is clarified in the figure legend. T-test and ANOVA one-way were used to evaluate significant differences between/among groups, using Prism software (GraphPad software). Data is shown as the mean ± standard error of the mean (SEM).

## Results

### AURKA is the only AURK that is necessary and sufficient for female fertility

In previous studies in which we deleted AURKA in oocytes, we showed that females deficient in AURKA are sterile because their oocytes arrest in metaphase I. We also showed that overexpression of AURKB or AURKC cannot rescue the oocyte defects (Blengini et al., 2021a). In contrast, females expressing only AURKA (BC-KO) in their oocytes, produce pups and their oocytes complete meiosis I and progress to meiosis II. Completion of meiosis I occurs because AURKA can bind the chromosomal passenger complex (CPC) and compensate for the loss of the other two isoforms (Nguyen et al., 2018). We endeavored to complete our assessment of genetic compensation and interactions. To this end, we generated a series of knockout mice in which we deleted the Aurora kinases in such a way that the oocytes express only AURKB (AC-KO); only AURKC (AB-KO); harbor only one copy of *Aurka* (A-Het, BC-KO) or a triple KO where no isoform is present in the oocytes (ABC-KO). Each single *Aurk* KO, the AB-KO and the ABC-KO mice have been validated previously (Blengini et al., 2021a; Blengini et al., 2024; Nguyen et al., 2018; Schindler et al., 2012). To confirm that the AC-KO and A-Het, BC-KO oocytes express only one AURK, we measured localized AURK activity of the remaining Aurora kinase by immunostaining with a pan-antibody that recognizes the three phosphorylated isoforms of Aurora kinase (pABC). In WT oocytes, pABC signal localized predominantly to spindle poles, which reports active AURKA, and to chromosomes, which reports active AURKC. In AC-KO oocytes, pABC signals were weak, mostly cytoplasmic, consistent with dispersed AURKB activity (Supplementary Figures S1A, C). In A-Het, BC-KO oocytes, pABC signal was detected mostly at spindle poles but also weakly on chromosomes, consistent with AURKA-CPC interaction in the absence of AURKC (Supplementary Figures S1B, D).

**FIGURE 1:**
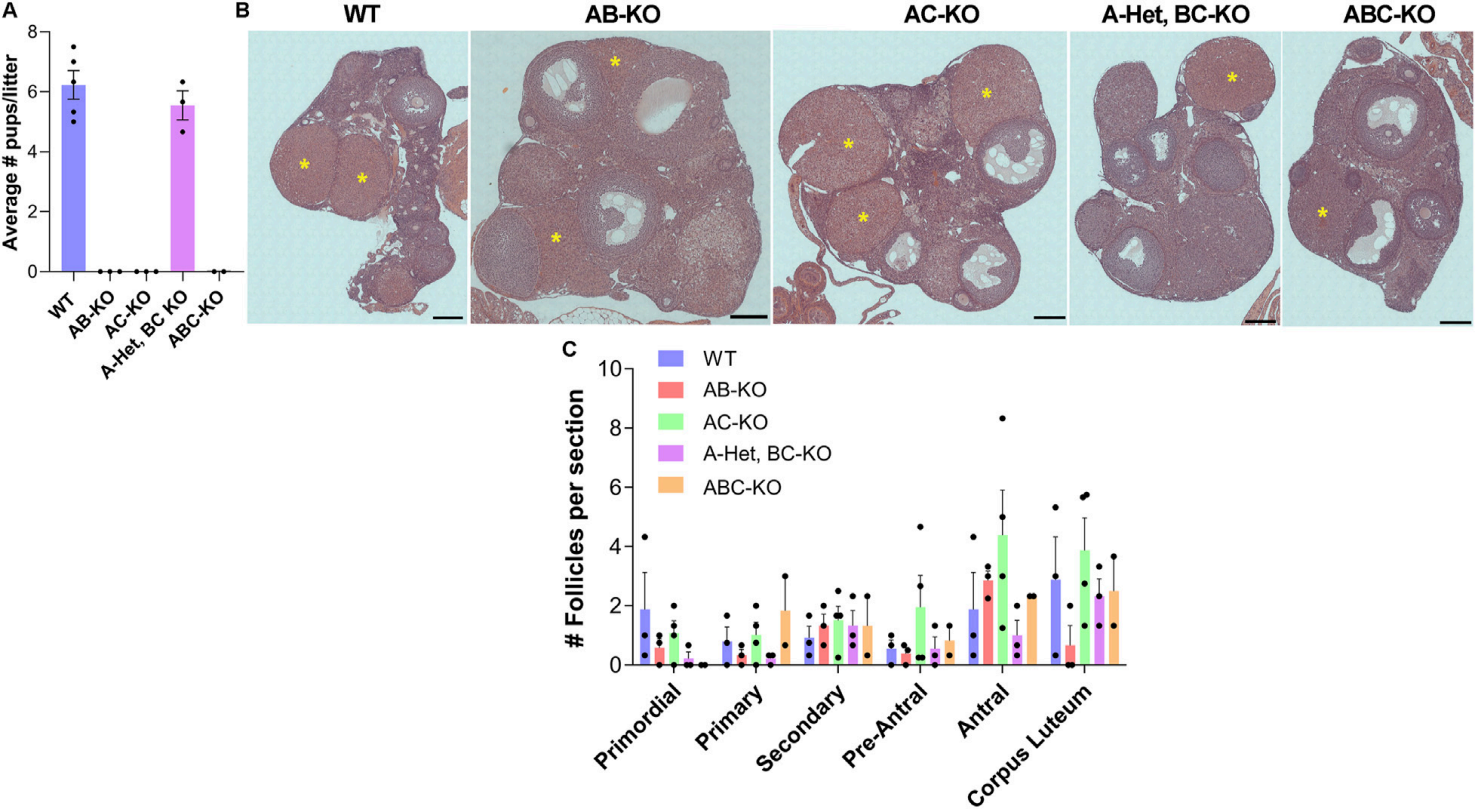
One copy of *Aurka* is required for female fertility. **(A)** Average number of pups per litter from females of indicated genotype. Two-way ANOVA: genotype factor *p* = 0.1544. **(B)** Representative images of hematoxylin/eosin-stained ovarian sections after fertility trials from females of the indicated genotypes. yellow asterisks: corpus luteum (CL). **(C)** Quantification of follicle types from the ovaries represented in **(B)**. Follicle numbers were quantified for each ovary and reported as the average number of each type of follicles per section; (2–4 females/genotype). One-way ANOVA for each type of follicle comparing the 5 genotypes: primordial *p* = 0.3345; primary *p* = 0.2708; secondary *p* = 0.9317; early antral *p* = 0.4804; antral *p* = 0.2922; and CL *p* = 0.3272. Scale bar: 200 μm.

To determine the physiological effect of deleting combinations of *Aurk*s from oocytes, we performed fertility studies by mating WT or KO females with WT males of robust fertility (Figure 1A). In contrast to WT females, which produced an average of 6 pups per litter (n = 5), the AB-KO (n = 3), AC-KO (n = 3) and ABC-KO (n = 2) females were sterile; no pups were observed. Surprisingly, A-Het, BC-KO females (n = 3) were fertile, producing an average of 5 pups per litter. This litter size was not significantly different from WT. Therefore, our studies confirm that AURKA is the only essential AURK for female fertility and suggest that the oocyte can tolerate reduced AURKA levels.

**FIGURE 2:**
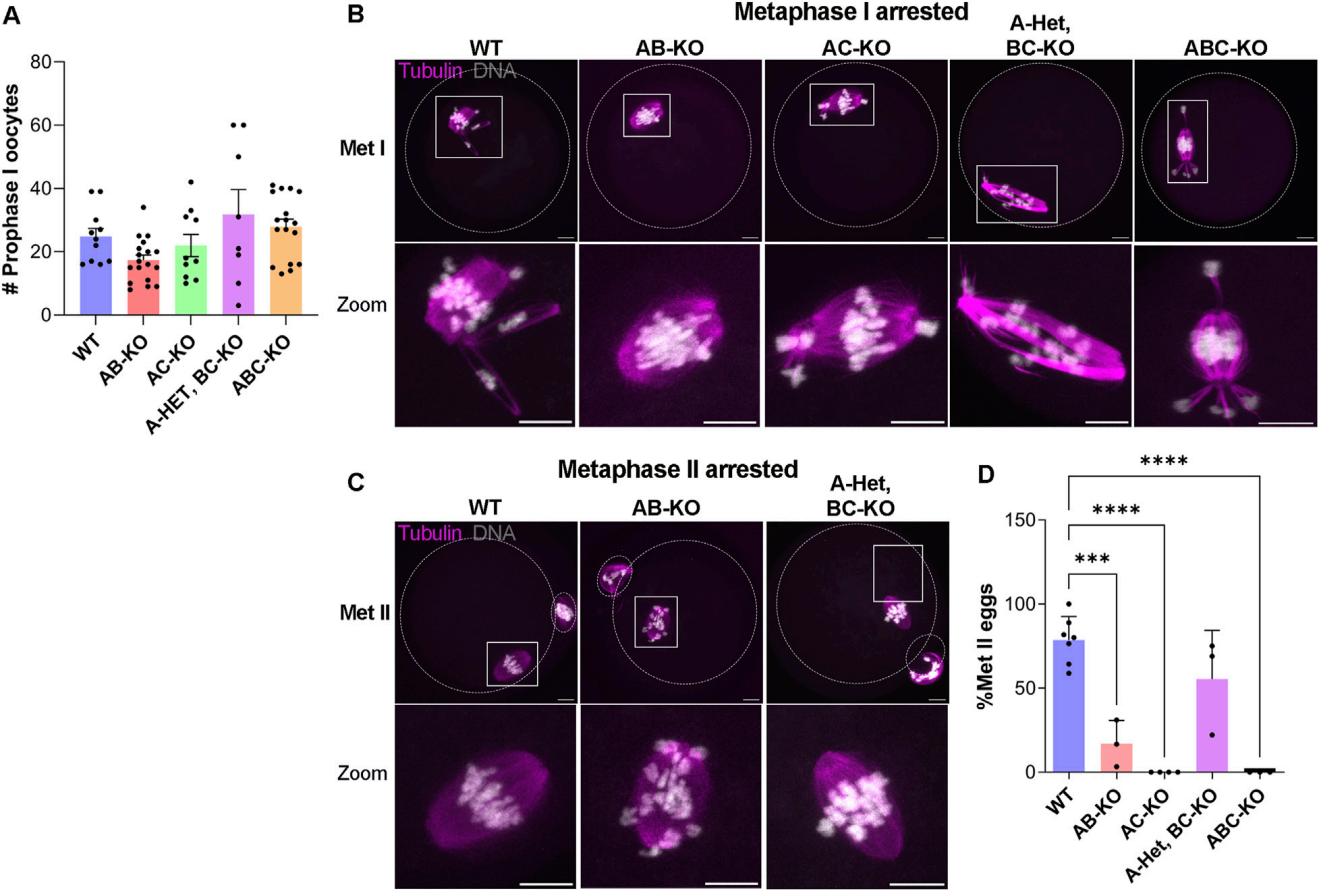
One copy of *Aurka* is required for completion of meiosis I *in vivo* **(A)** Quantification of the number of prophase-I arrested oocytes retrieved from females with the indicated genotypes (# of females WT: 11, AB-KO:18; AC-KO:10, A-Het, BC-KO: 8; ABC-KO: 17) (One-way ANOVA comparing to WT: AB (*p* = 0.4374), AC (*p* = 0.9798), A-Het, BC-KO (*p* = 0.6908), ABC-KO (*p* = 0.9576)). **(B,C)** Representative confocal images of ovulated oocytes immunostained with Tubulin (pink) and DNA (gray) arrested in metaphase I **(B)** and arrested in metaphase II **(C)**. The dotted outlines indicate the oocyte and polar body membranes. Scale bars: 10 μm. **(D)** Quantification of the percentage of Met II eggs retrieved from females with the indicated genotypes (# of females WT: 7, AB-KO: 3; AC-KO: 4, A-Het, BC-KO: 3; ABC-KO: 3). (One-way ANOVA, *p < 0.05; ***p < 0.001; ****p < 0.0001).

We then investigated the potential causes of female infertility. After fertility trials, we collected ovaries for histological analyses. These females generated all follicle types without significant differences among genotypes (Figures 1B, C). Importantly, corpus lutea (CL) were present in histologic sections indicating that sterility was not due to ovulation defects (Figures 1A, B). We note that we were not able to detect many primordial follicles from these animals. This result is likely because the animals were retired from the fertility trial and therefore they were 8–9 months of age, an age where the ovarian reserve starts to decline. The numbers of prophase I arrested oocytes isolated from females aged 6–12 weeks were comparable to WT (Figure 2A). AC-KO and ABC-KO females ovulated oocytes arrested in metaphase I with 100% displaying severe chromosome misalignment (AC-KO: 70 oocytes; ABC-KO 41 oocytes); oocytes from WT females occasionally were defective (Figures 2B, D). In contrast to all other strains lacking *Aurka* that arrest in MI, a small percentage of AB-KO oocytes reached metaphase II (Figures 2C, D). We retrieved 16.9% (12/71 oocytes) MII eggs after hormonally stimulating ovulation and 6.7% (2/30 oocytes) MII eggs after *in vitro* maturation. The ability to undergo cytokinesis despite severe chromosome and spindle abnormalities (Figure 2C) suggests a failure to arrest via the spindle assembly checkpoint. Consistent with the fertility data, 60% of oocytes from A-Het BC-KO females completed meiosis I *in vivo*, and ovulated metaphase II arrested eggs with normal chromosome alignment and spindle morphology (Figures 2C, D). These results indicate that only one genomic copy of *Aurka* is sufficient to complete meiosis I in mouse oocytes and suggest that oocytes that express AURKC alone have defects in the spindle assembly checkpoint.

### One copy of *Aurka* is required for spindle formation

**FIGURE 3:**
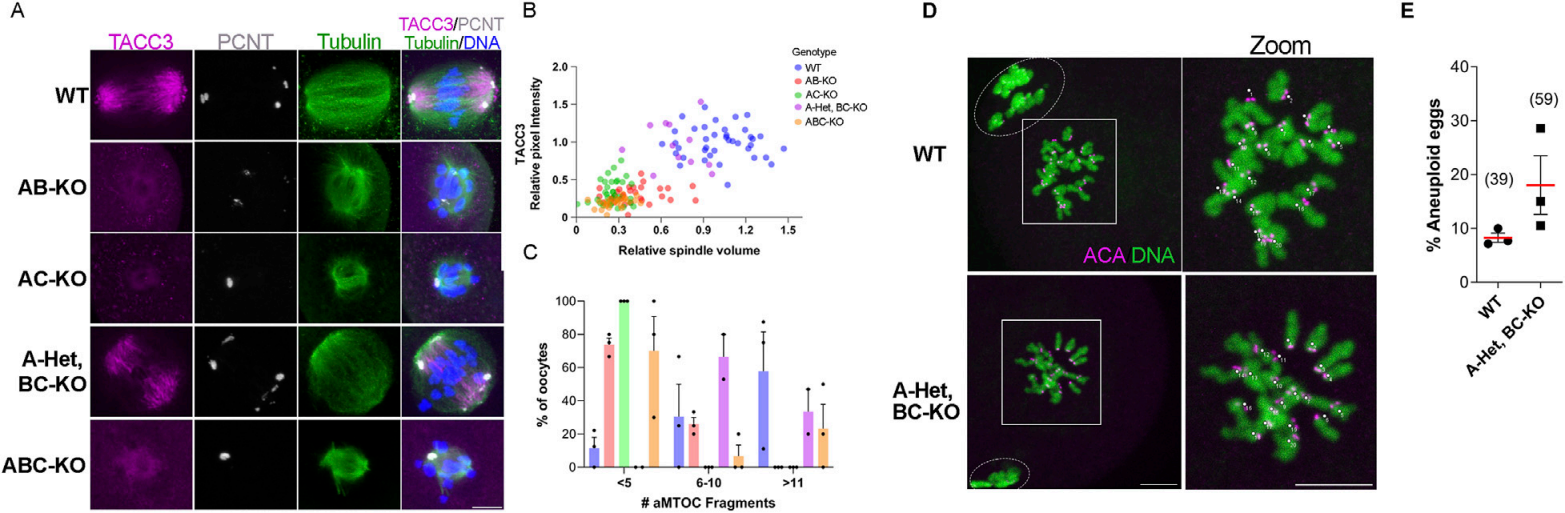
One copy of *Aurka* is required for MI spindle assembly and euploid egg production. **(A)** Representative confocal images of metaphase I oocytes from females of the indicated genotypes immunostained with TACC3 (pink), PCNT (gray), Tubulin (green) and DNA (blue). Scale bar: 10 μm. **(B)** Quantification of spindle volume and TACC3 intensity in **(A)**. **(C)** Quantification of the percentage of oocytes with a determined number of aMTOC fragments in **(A)**. See also Supplementary Figure S2 which contains statistics. **(D)** Representative confocal images of *in situ* chromosome spreads of metaphase II eggs from females of the indicated genotypes immunostained with ACA (gray) and DNA (blue). The numbers (pink) indicate the sister chromatid pair. Scale bar: 10 μm. **(E)** Quantification of aneuploid eggs in **(D)**. 3 female per genotype. (Two tailed T-test, *p* = 0.1508).

To understand AURK requirements in oocyte maturation, we further evaluated A-Het, BC-KO oocytes using them as a simplified and sensitized genetic background. Because AURKA is required for multiple steps of spindle building, we evaluated three spindle parameters at metaphase I. We measured spindle volume, intensity of TACC3, as a readout of LISD, and aMTOC numbers around the spindle. When spindle volume and TACC3 intensity were considered together, a gradient of phenotypes was observed. At the extremes, WT oocytes had larger spindle volumes and higher TACC3 intensities whereas AB-KO, AC-KO, and ABC-KO oocytes had smaller spindle volumes and lower TACC3 intensities. We observed an intermediate phenotype in A-Het, BC-KO oocytes which had smaller spindles compared to WT but had comparable levels of TACC3 (Figure 3A, B; Supplementary Figures S2A, B). It is important to highlight that the TACC3 domain is disorganized in the absence of AURKA, as evidenced by the increase in TACC3 background signal in these oocytes (Figure 3A). This result is consistent with previous data suggesting that AURKA is essential for TACC3 organization in mouse oocytes (Blengini et al., 2021a; Blengini et al., 2024; So et al., 2019). According to past studies reporting the role of AURKA in maintaining spindle pole structure, the majority the oocytes from WT and A-Het BC-KO animals, had more than six aMTOC fragments around the spindle (Figure 3C) compared to oocytes from AB-KO, AC-KO, and ABC-KO females where most oocytes had fewer (less than 5) (Figure 3C). Taken together, these data indicate that only one genomic copy of *Aurka* is necessary and sufficient for spindle pole organization, LISD formation, and thus spindle structure at metaphase I in mouse oocytes.

### One copy of *Aurka* is sufficient to produce euploid eggs

Because A-Het, BC-KO females were fertile and their oocytes produced relatively normal bipolar meiosis I spindles, the next step was to evaluate levels of egg aneuploidy. Therefore, we matured WT and A-Het, BC-KO oocytes for 16h until they reached metaphase II, after which we conducted *in situ* chromosome spreads to count the number of sister chromatid pairs. A-Het, BC-KO females produced a slightly higher percentage of aneuploid eggs than WT, although this rate was not significant (Figures 3D, E). These data suggest that only one genomic copy of *Aurka* is necessary to support proper chromosome segregation during meiosis I.

## Discussion

In this study, we completed an oocyte-AURK genetic interaction map, and show that one genomic copy of *Aurka* is necessary and sufficient to support female fertility and oocyte meiosis. We further confirm and that AURKB and AURKC alone cannot compensate for AURKA. These results highlight the importance of AURKA in mouse oocytes, demonstrating that it is required for spindle formation and proper chromosome segregation. Why is AURKA the only Aurora kinase that is required for oocyte meiosis? First, in evolutionary terms, primitive organisms such as yeast possess only one isoform of AURK, *IPL1*, which is phylogenetically more similar to mouse AURKA than the other 2 mouse isoforms (Brown et al., 2004; Radford et al., 2017; Willems et al., 2018). This analysis reveals that evolutionarily AURKA has the potential to perform all the functions required for meiosis in the absence of AURKB/C, by being flexible to adapt to different binding partners and substrates. Second, based on the strong phenotype of oocytes lacking AURKA and the ability of a single copy to rescue oocyte quality, we speculate that AURKA is involved in several critical cellular mechanisms for oocyte meiosis besides spindle formation (Blengini et al., 2021a; Blengini et al., 2024). For example, there is evidence in several model systems that AURKA is important for protein homeostasis by regulating translation (Aboelenain and Schindler, 2021; Han et al., 2017; Kunitomi et al., 2024) and protein degradation (Briassouli et al., 2006; Fang et al., 2023). Other studies show it is involved in mitochondrial dynamics and function (Bertolin et al., 2021; Bertolin et al., 2018). However, whether AURKA is involved in these processes in mouse oocytes and the molecular mechanisms driving these functions requires further investigation.

**FIGURE 4:**
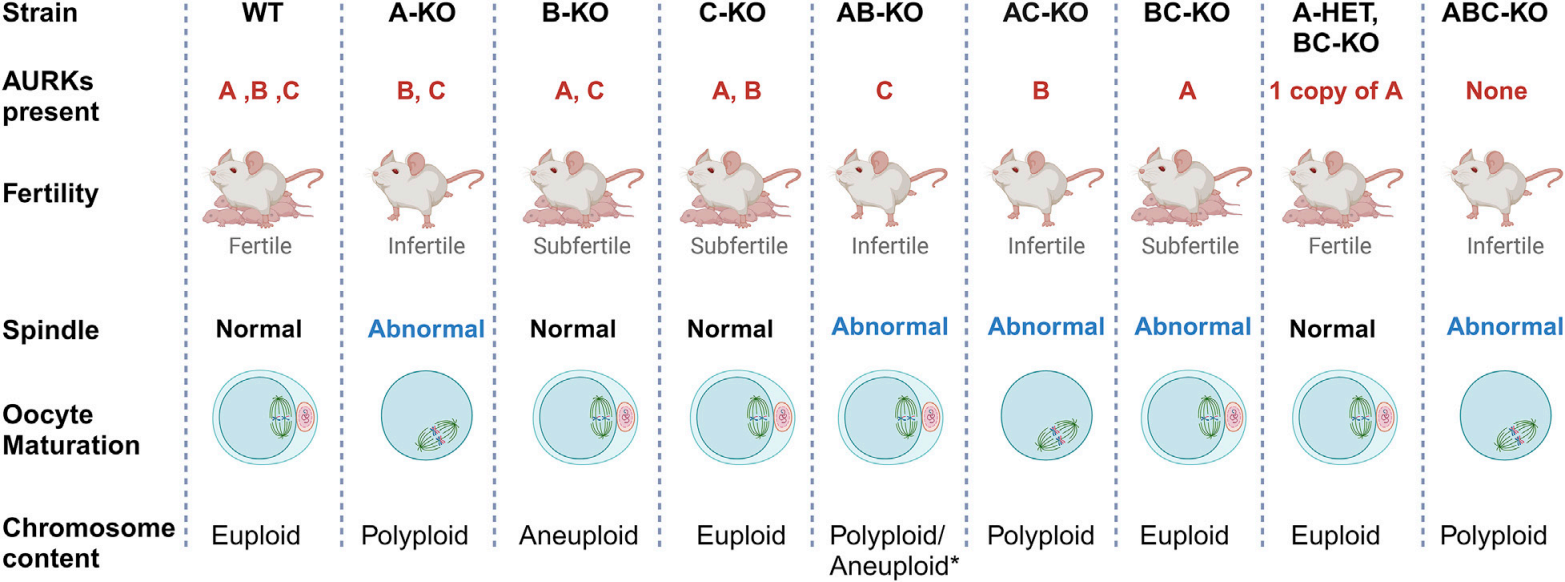
Summary of *Aurk* genetic interaction mapping. Schematic summary of the genetic interplay of Aurora kinases in mouse oocytes. The main phenotypes of females with different Aurora kinase content are presented. Most strains that lack of AURKA are sterile with abnormal meiotic spindle at metaphase I and failure to complete meiosis I. As a result, they produce polyploid oocytes (Blengini et al., 2021a). * AB-KO oocytes are the exception, a small percentage complete meiosis I and produce eggs likely aneuploid based on severe spindle phenotypes. B-KO females are sub-fertile, producing high rate of aneuploid egg with maternal aging (Blengini et al., 2021b; Nguyen et al., 2018). C-KO females are also sub-fertile (Schindler et al., 2012).

Because AURKA is the sole essential AURK, a question arises of why mouse oocytes express AURKB and AURKC. Collectively, our previous work demonstrates that there is a fitness advantage to expressing the three isoforms (Figure 4). Mice with single *Aurkb* and *Aurkc* deleted oocytes are subfertile, having smaller than average litter sizes (Nguyen et al., 2018; Schindler et al., 2012). The causes for subfertility differ. We speculate that the levels of localized activities of each AURK are important for oocyte maturation and egg quality. Specifically, mice lacking *Aurkb* in their oocytes undergo age-related declines in fertility, likely because AURKB is required to limit AURKA and AURKC activity (Aboelenain and Schindler, 2021). The phenotypes of *Aurkb* knockout oocytes phenocopy those with overexpressed AURKA (increased translation) (Aboelenain and Schindler, 2021) or AURKC (SAC evasion) (Sharif et al., 2010). Furthermore, AURKA gains chromosomal localization in oocytes lacking AURKC without a significant increase in total AURKA protein, suggesting that AURKC is required to ensure sufficient AURKA activity at aMOTCs (Nguyen et al., 2018).

Surprisingly, a small percentage of AB-KO oocytes completed meiosis I and expelled polar bodies. However, these females were sterile, suggesting that egg quality was compromised, a phenotype that we could not assess due to the severity of the MII spindles. Our previous studies showed that the spindle assembly checkpoint (SAC) is weak in AURKB deficient oocytes (Blengini et al., 2021b). To evaluate SAC strength, one must use oocytes prior to anaphase I onset. This means that a cohort of oocytes used for this analysis are heterogenous for SAC defects. Only 6% of AB-KO oocytes from 1 mouse extrude polar bodies when matured *in vitro*. The average oocyte sample size is 20; therefore, changes in MAD2 levels in one or two oocytes would not be significant enough to detect. Therefore, it is technically difficult to detect changes in the levels of SAC components in those oocytes that would go on to complete meiosis I. In B-KO oocytes, AURKC activity is elevated and overexpression of AURKC in WT oocytes causes advanced APC/C activation (Nguyen et al., 2018; Sharif et al., 2010). Therefore, we speculate that SAC failure and/or cell cycle dysregulation is the reason why some AB-KO oocytes expel polar bodies. In conclusion, using a comprehensive genetic mapping approach, we wholly define the genetic interplay among the Aurora kinases (Figure 4) and reinforce the importance of AURKA in oocyte meiosis. Whether AURKA plays a similar dominate role in human oocytes is a question of high priority to answer.

## Data Availability

The raw data supporting the conclusions of this article will be made available by the authors, without undue reservation.

## Supplementary material

SUPPLEMENTARY FIGURE S1 Genotype confirmation of AC-KO and A-HET, BC-KO oocytes **(A)** Representative confocal images of metaphase I oocytes from WT and AC-KO females immunostained with ACA (pink), pABC (gray), Tubulin (green) and DNA (blue). Scale bar: 10 μm. **(B)** Quantification of pABC intensity (A) (t-test, ****p < 0.0001). Representative confocal images of metaphase I oocytes from WT and A-HET, BC-KO females immunostained with ACA (pink), pABC (gray), Tubulin (green) and DNA (blue). Scale bar: 10 μm. **(D)** Quantification of pABC intensity **(C)** (t-test, p = 0.4213). WT pABC intensity is the same used to validate AB-KO and ACB-KO in a previous publication because these experiments were conducted at the same time (Blengini et al., 2024). In brackets is the number of oocytes analyzed in at least 3 independent experiments.

SUPPLEMENTARY FIGURE S2 Statistical analyses from Figure 3. **(A)** Relative spindle volume and **(B)** TACC3 relative pixel intensity in metaphase I oocytes from the indicated genotypes. In brackets is the number of oocytes analyzed in at least 3 independent experiments. One-way ANOVA; *****p*-value <0.0001. Data are represented as mean ± SEM.
