# Supplementary figures and images for: Genetic interaction mapping of Aurora protein kinases in mouse oocytes
Source: Front Cell Dev Biol. 2024 Sep 25;12:1455280. doi: 10.3389/fcell.2024.1455280 (PMC11461192; doi:10.3389/fcell.2024.1455280)

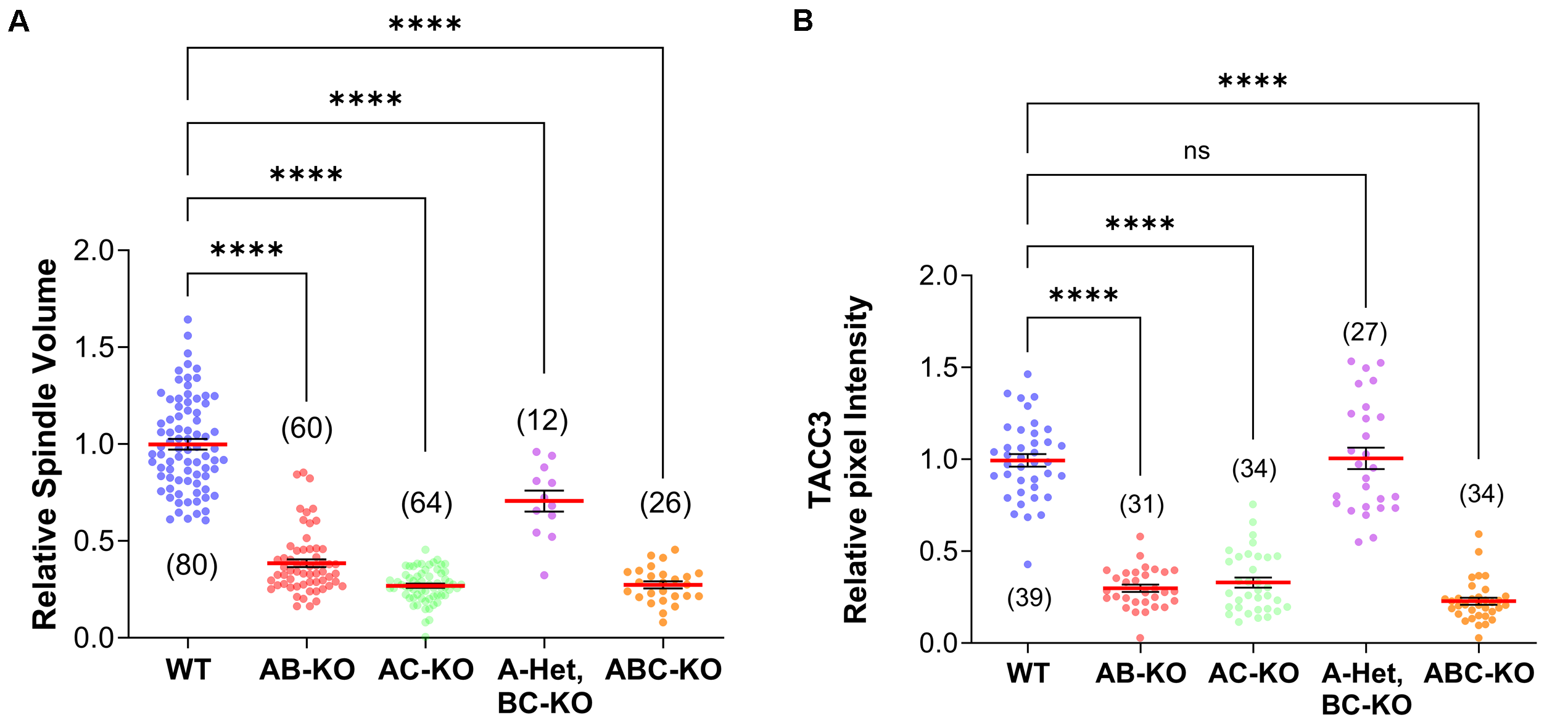

Supplement: Supplementary file 1 [file Image2.TIF]

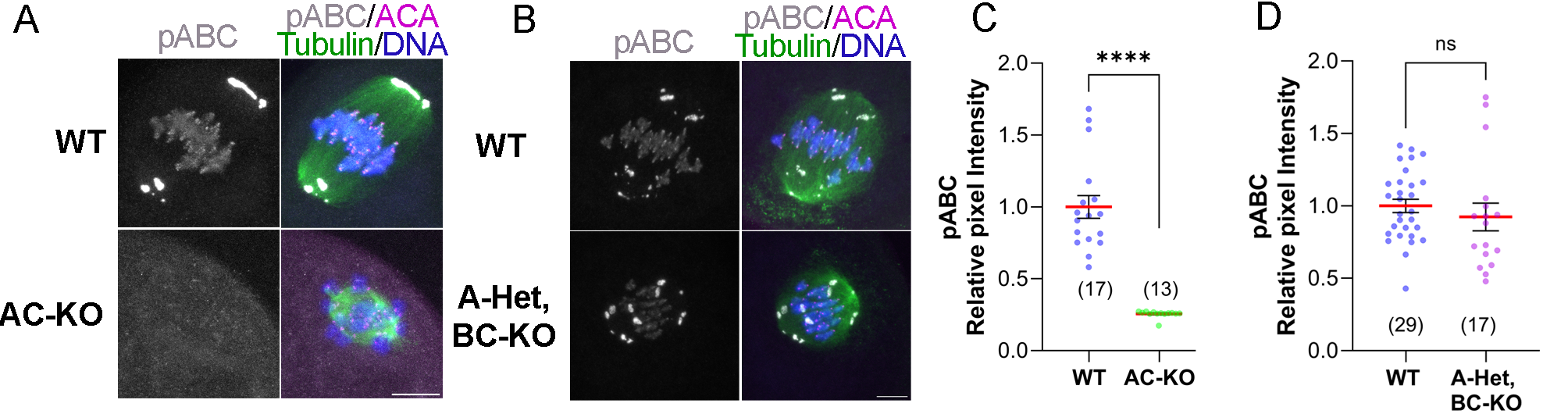

Supplement: Supplementary file 2 [file Image1.TIF]
